# Synergies between Urban Heat Island and Heat Waves in Athens (Greece), during an extremely hot summer (2012)

**DOI:** 10.1038/s41598-017-11407-6

**Published:** 2017-09-08

**Authors:** Dimitra Founda, Mattheos Santamouris

**Affiliations:** 1 National Observatory of Athens, Institute for Environmental Research & Sustainable Development, Athens, GR-15236 Greece; 2 The Anita Lawrence Chair in High Performance Architecture, School of Built Environment, University of New South Wales, Sydney, 2052 Australia; 3 Physics Department, University of Athens, Athens, GR- 15772 Greece

## Abstract

Heat waves (HWs) are recognized as a serious threat for human health worldwide, with urban areas being more vulnerable due to the urban heat island (UHI) effect and population density. Yet, in the climate change context, HWs are becoming more frequent, stronger and longer, which, coupled with intensifying urbanization exacerbates thermal risk for urban residents. Despite the profound impact of this global phenomenon there is no clear consensus so far on possible synergies between UHIs and HWs. The study sheds light on the complex synergies between UHIs and HWs focusing on coastal sites. A quite challenging period comprising five HW episodes during summer 2012 in Athens (Greece) was selected for analysis. A positive feedback between UHIs and HWs was found, with intensification of the average UHI magnitude by up to 3.5 °C during HWs, compared to summer background conditions. Our results contribute significantly to understanding synergies between UHIs and HWs that may strongly increase thermal risk in cities and vulnerability of urban population.

## Introduction

The global environment is characterized by profound changes, of which intensifying urbanization and climate change are among the most momentous. Today, more than half of the world’s population lives in urban areas and the proportion is projected to increase further in the near future^1^.

Urban regions experience hotter conditions compared to their natural surroundings, due to a number of factors that modulate urban climate and form ‘Urban Heat Islands’ (UHIs)^2^. The main contributing factors are changes in the surface energy budget due to increased heat storage capacity of artificial surfaces compared to natural ones, reduction of evaporative cooling, differences in convective and advective flows and increased anthropogenic heat release in urban areas^3, 4^. The Urban Heat Island phenomenon is documented in more than 400 cities around the world, and is responsible for a considerable increase in cooling energy consumption as well as exacerbation of air pollution, while it constitutes a serious threat for human health^5–7^. Its intensity is quantified on the basis of the temperature difference between urban and adjacent non urban sites^2^.

At the same time, global warming forcing has resulted in more frequent, severe and longer lasting excessive heat events (heat waves) worldwide, while there is strong evidence that the frequency and severity of such extreme events will increase further in the near future^8–14^. In addition to the environmental and economic impacts, heat waves have a quite devastating impact on human health and in many parts of the world kill more people than any other natural hazard^4, 15–19^. Duration, intensity and timing of HWs, but mostly societal vulnerability (e.g. age, health condition, poverty or isolation) have been proven to influence thermal risk and mortality^20–23^. It becomes clear that the adverse effects of HWs are more pronounced in urban areas due to the higher population density and the potential additive effect of the UHI^24–29^. As a result, severe heat waves have led to numerous excess deaths in many cities of the world in the recent past (e.g. Athens, 1987^30^; Chicago, 1995^31^; Paris, 2003^32^; Adelaide, 2009^33^; Moscow, 2010^34^).

Taking into account the observed and projected increasing trends in both urban population and HWs frequency and -the most important- the well established relationship between HWs and public health, it is expected that urban residents will very likely be exposed to particularly enhanced thermal risk in the near future^16, 26^. Thus, understanding the interactions and possible synergies between UHIs and HWs in the modulation of urban thermal conditions is of vital importance.

The subject has received much attention recently; nonetheless, findings are not always convergent and there is no clear consensus so far on how UHIs respond under exceptionally hot weather. In particular, simulations and future projections on the possible impact of global warming on the magnitude of UHIs are quite contradictory, indicating either reduction^35^, or no significant modification of UHI intensity under intensified climate change conditions^36^. Recent research suggests synergistic reactions between UHIs and HWs and stronger impact in cities, more pronounced during nighttime^37–41^. According to other studies, HWs induce significant but mixed modifications in the spatial pattern of the urban heat island during daytime^42^. Background local climates have been found to strongly contribute to synergies between UHIs and HWs, with exacerbation of HW stress in wet climates during dry years^43^. A reverse reaction to global warming that induces cooling at coastal areas during summer days is also reported^44^.

The present study aims to provide important additional scientific information to enrich the existing knowledge and further enlighten the characteristics of this phenomenon. To this end, it analyses experimental data obtained under exceptionally hot conditions at a large coastal urban area of the eastern Mediterranean, Athens. The Mediterranean has been identified as one of the regions most vulnerable to climate change and moreover as a ‘hotspot’ in terms of future thermal risk^14, 45^. Besides, coastal areas stand at the focus of attention as they are expected to host almost 75% of the global population by 2025, while thirteen of the world’s 20 megacities lie along coasts^46^.

A challenging period marked by successive HWs and variable background synoptic conditions was selected for analysis. Four coastal stations (CS1-CS4) and the reference urban station of the National Observatory of Athens (NOA) were used for the estimation of the UHI intensity (See Methods).

For the first time, to our knowedge, the synergetic action of UHI and HWs has been considered at coastal sites accounting for alterations in surface energy budget and horizontal advection phenomena. Sea breeze circulations, acting either in competitive or in synergistic roles with synoptic winds, were found to strongly impact the daytime UHI intensity during HWs, while radiative processes determined the magnitude of the nocturnal UHI.

## Results

**Figure 1:**
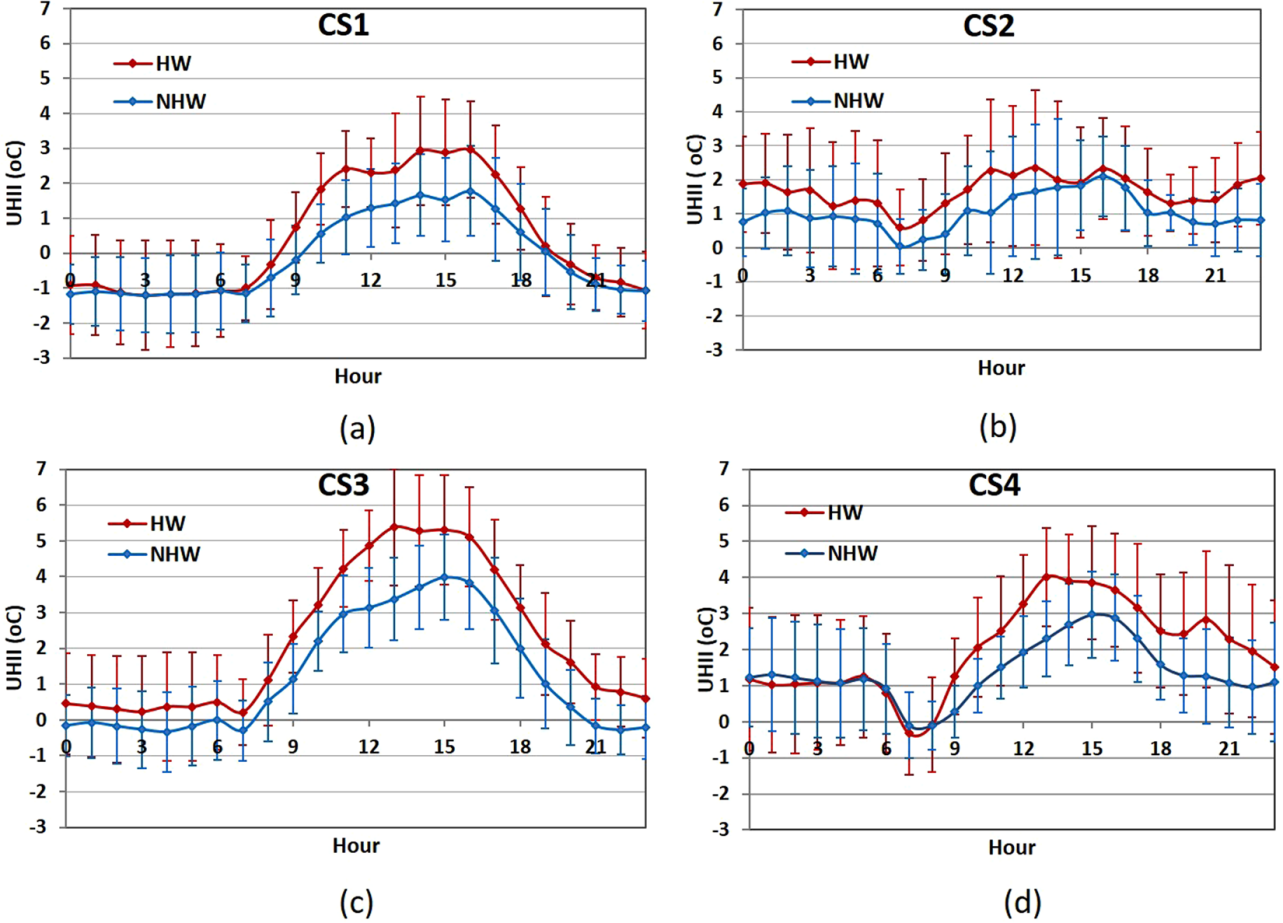
**(a–d**) Diurnal patterns of average UHI intensity along with standard deviations at CS1-CS4, under HW and NHW conditions, over the study period (July-August 2012).

The UHI magnitude was calculated during the study period at all stations. Figure 1(a–d) depicts the mean diurnal pattern of the UHII at the four stations CS1-CS4, separating for NHW and HW conditions. As can be seen in the figure, the magnitude of UHI reveals a strong diurnal variation, with positive and higher values occurring during daytime and in particular during noon and afternoon hours. It is notable that the pattern deviates from characteristic diurnal UHI patterns, given that the UHI is typically viewed as a nighttime phenomenon in the literature. This can be attributed to local circulations and more steady sea surface temperatures prevailing at coastal sites (compared to rural ones) and is further analysed below.

A significant amplification of the strength of the UHI is observed during HW compared to NHW periods in all cases (Fig. 1). The amplification is higher during daytime, except for CS2, where large differences are also observed during nighttime. In addition to the diurnal variation, UHII values also vary considerably around their means, at all sites. Additional analysis revealed a strong dependence of the daytime UHI intensity on the daily maximum ambient temperature (T_*max*_) and the wind speed and direction at the reference site, which is discussed below.

### Daytime UHI

**Figure 2:**
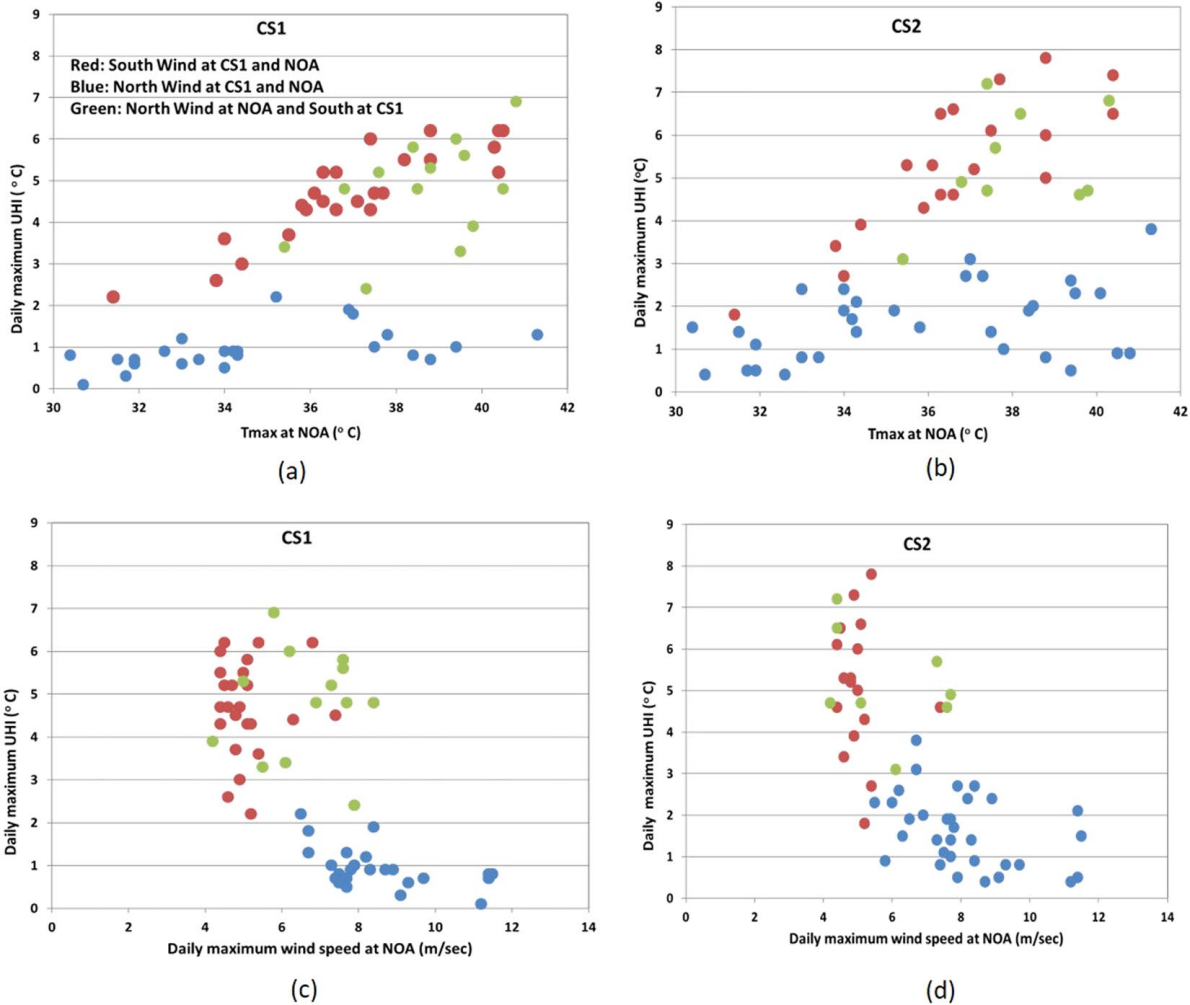
**(a–d**) Variation of the daily maximum UHI intensity at CS1 and CS2 with T_*max*_ (**a**,**b**) and daily maximum wind speed (**c**,**d**) at NOA, aggregating for 3 categories (combinations) of wind direction: i) northerly winds (advection from mainland) at both NOA and coastal station (blue color), ii) southerly winds (sea breeze) at both NOA and coastal station (red color) and iii) northerly winds at NOA and southerly winds at coastal station (green color).

During the study period, daily maximum wind speed at the reference station ranged between 4.4 and 11.5 m/sec during NHW and between 4.2 and 8.4 m/sec during HW periods. The most frequent wind sectors were the Northeastern (NE), corresponding to the ‘Etesians’ synoptic pattern and Southwestern (SW), corresponding to sea breeze circulation (see Methods). We found that the UHI amplitude is highly determined from the wind speed and direction but also the daily maximum ambient temperature (T_*ma*x_) at NOA. Figure 2(a–d) presents the daily maximum UHII at CS1 and CS2 as a function of T_*max*_ and the daily maximum wind speed at NOA. Note that the daily maximum UHII and the daily maximum ambient temperature are not necessarily synchronized, although they usually occur around afternoon hours. According to Fig. 2, the magnitude of UHII increases with increasing temperature and in general decreases with increasing wind speed. (The pattern is consistent at all four stations, as also shown in Fig. S4 in Supplementary). However, the pattern changes considerably with changing wind direction and in particular, when the wind blows: a) from northern directions at both the reference and coastal sites, b) southern directions at both the reference and coastal sites and c) northern direction at the reference and southern at coastal sites. Actually, the synoptic wind largely determined the development or not of sea breeze at the coastal sites, influencing the amplitude of UHI. As shown in Fig. 2(c,d) strong winds (>8 m/sec) correspond always to northern directions at all sites (suggesting the dominance of the ‘Etesians’ synoptic pattern), blocking the development of sea breeze circulations and keeping UHII at minimum. Attenuation of ‘Etesians’ (wind speed approximately lower to 8 m/sec) enables occasionally the development of sea breeze at coastal sites (southerly winds at the coast), although northerly winds may still prevail at the urban site. Finally, in the absence of the ‘Etesians’ pattern, southerly winds prevail at both urban and coastal sites. The last two patterns, associated with the onset of sea breeze circulation at the coastal stations, maximize the UHI intensity (Fig. 2).

The above classification of the wind patterns applies in a somewhat different way at the coastal stations CS3 and CS4, due to the different direction of the sea breeze (see Fig. S1 in Supplementary). This means that synoptic northeasterly winds correspond to advection from the sea at these stations and in such cases, sea breezes and synoptic winds may act cooperatively, further increasing the cooling mechanism at the coastal stations and maximizing UHI intensity.

**Figure 3:**
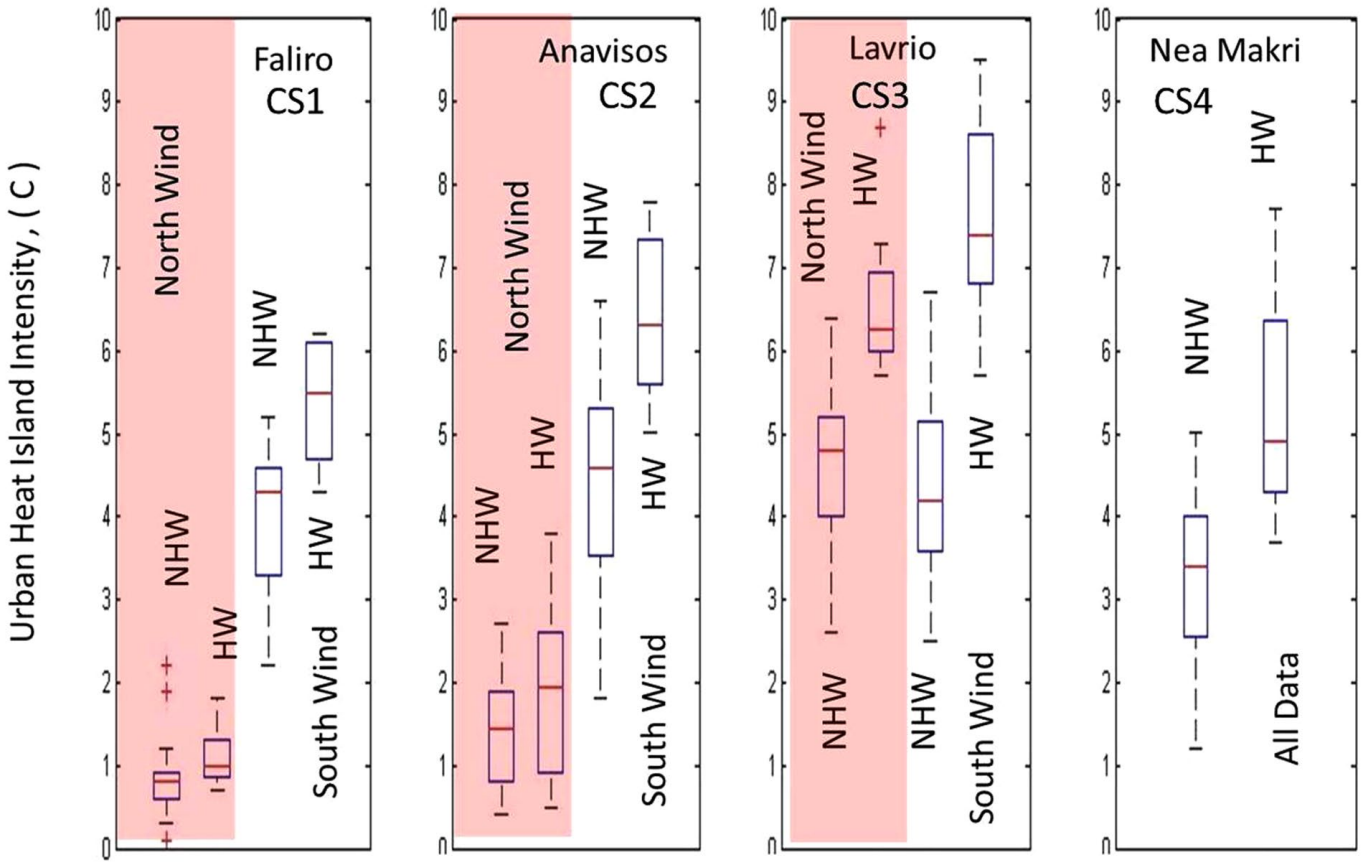
Boxplots of hourly values of daytime UHI intensity at all stations (CS1-CS4), during HW/NHW periods and under southern/northern wind directions at the reference station.

Figure 3 illustrates boxplots of the daily hourly values of UHI intensity at all coastal stations (CS1-CS4), separating the specific cases of HW/NHW conditions and advection from the mainland/sea (northern/southern winds respectively) at the reference station. A first conclusion derived from the figure is that southerly winds are in general associated with higher UHI intensity compared to northerly (and stronger) winds at all stations, during both HW and NHW periods. A second conclusion is that HW periods are associated with higher UHI intensity (compared to NHW periods) at all stations under both southerly and northerly winds. The highest UHI intensities are observed during HWs and under southerly winds. According to Fig. 3, under southerly winds, UHI is intensified by 1–3.5 °C (depending on the station) during HWs, compared to NHW periods. Under northerly winds, UHI intensification during HWs is less prominent, reaching up to 1.5 °C. UHII at station CS1 is less intensified by HWs compared to the other coastal stations, due to the higher urban density and its location, resulting to the advection of warm air from the city in the case of northerly winds. The most prominent increase of UHII during HWs is observed at CS3. Under southerly winds, the average increase of UHII approximates 3.5 °C at this station, while it may also exceed 8 °C.

The above analysis highlights the role of the wind field on daytime UHI intensity during HWs. In the following, we further analyse changes in the land surface temperature and near-surface humidity (associated with sensible and latent heat fluxes respectively), given their important roles in the surface energy balance equation.

The ground surface temperature increases during heat waves. Higher surface temperatures alter the incoming radiation partitioning between the components of the surface energy budget equation, such as evaporation rates, sensible heat flux and ground stored energy^39, 40^ (e.g. equation 2 - Methods). Because of the higher density and the increased thermal capacitance of cities, heat absorption and storage is considerably higher in urban than in rural areas. During HWs, ground surface temperature is substantially higher in the urban than in the rural areas, as well^47^. In urban areas, it may increase the released sensible heat and reduce the latent heat proportionally higher than in the rural areas, resulting to amplification of the surface temperature related urban heat island effect^39^.

**Figure 4:**
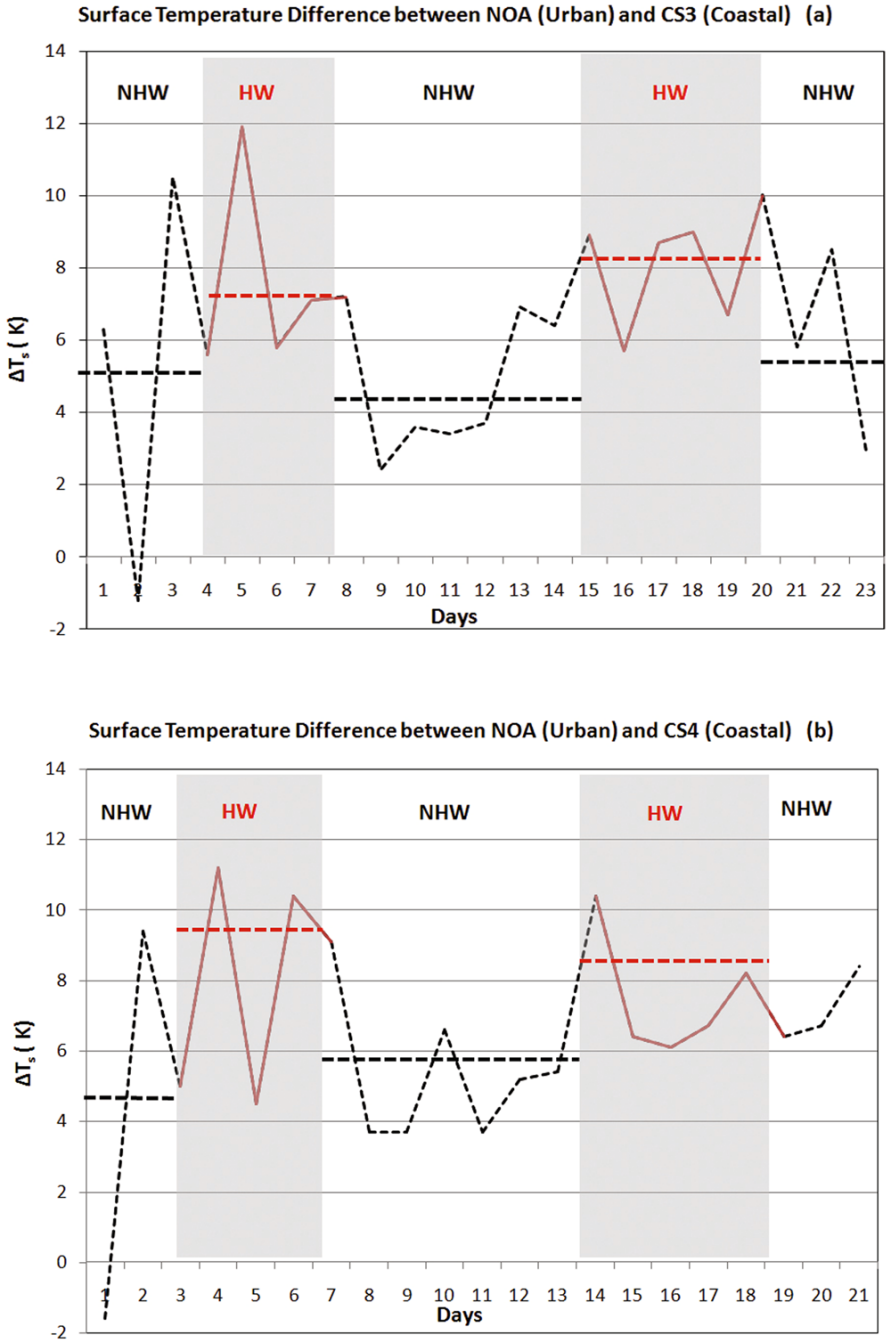
**(a,b)** Surface temperature difference between the urban site (NOA) and the coastal stations CS3 (**a**) and CS4 (**b**), during HWs and the preceding and following NHW periods. (The NHW periods correspond to 1, 3, 4, 14, 16–18, 20, 21 and 28–30 of August 2012 at CS3 and 3, 4, 14, 16–18, 20, 21, 28 and 29 of August 2012 at CS4, depending on availability of satellite data. The HW periods correspond to 5–9 and 22–27 of August 2012 at CS3 and CS4).

To investigate the relative changes in the surface temperature between HW and NHW periods at both urban and coastal stations, satellite daily surface temperature data derived from MODIS retrievals were used (see Methods). Surface temperature data were analysed for the HWs and the preceding and following periods (NHWs). The calculated daily surface temperature difference between the urban and the coastal stations CS3 and CS4 under HW and NHW conditions is shown in Fig. 4(a,b). During HWs, the surface temperature at NOA was found to increase by 3.0–3.5 °C compared to the preceding and following NHW periods. The corresponding increase at the coastal stations was lower and varied between 1.0 to 2.0 °C. The average surface temperature difference between the urban and coastal stations during the NHW periods was between 3.9 to 5.7 °C, while it increased and ranged between 7.5 to 8.2 °C during the HWs (Fig. 4a,b). A similar increase in the difference between the urban and rural surface temperatures during HWs has been also reported in Baltimore^39^. The surface temperature difference was significantly lower in Baltimore than in the present study -up to 4 °C- mainly because of the lower incident solar radiation. Simulation studies have shown that even a difference of 4 °C in the surface temperature during HWs, may result to increased sensible heat release in the urban compared to the rural areas, intensifying the urban heat island intensity^39^.

Differences in latent heat fluxes between urban and rural sites are also expected during HWs. Higher surface temperatures increase evapotranspiration from the ground to the atmosphere and the released latent heat contributes to the reduction of the ambient temperature. Evapotranspiration rates depend on the availability of the ground surface moisture and vegetation. Rural areas exhibit a higher availability of surface moisture than urban zones and present a higher potential for evapotranspiration and reduction of the ambient temperature.

**Figure 5:**
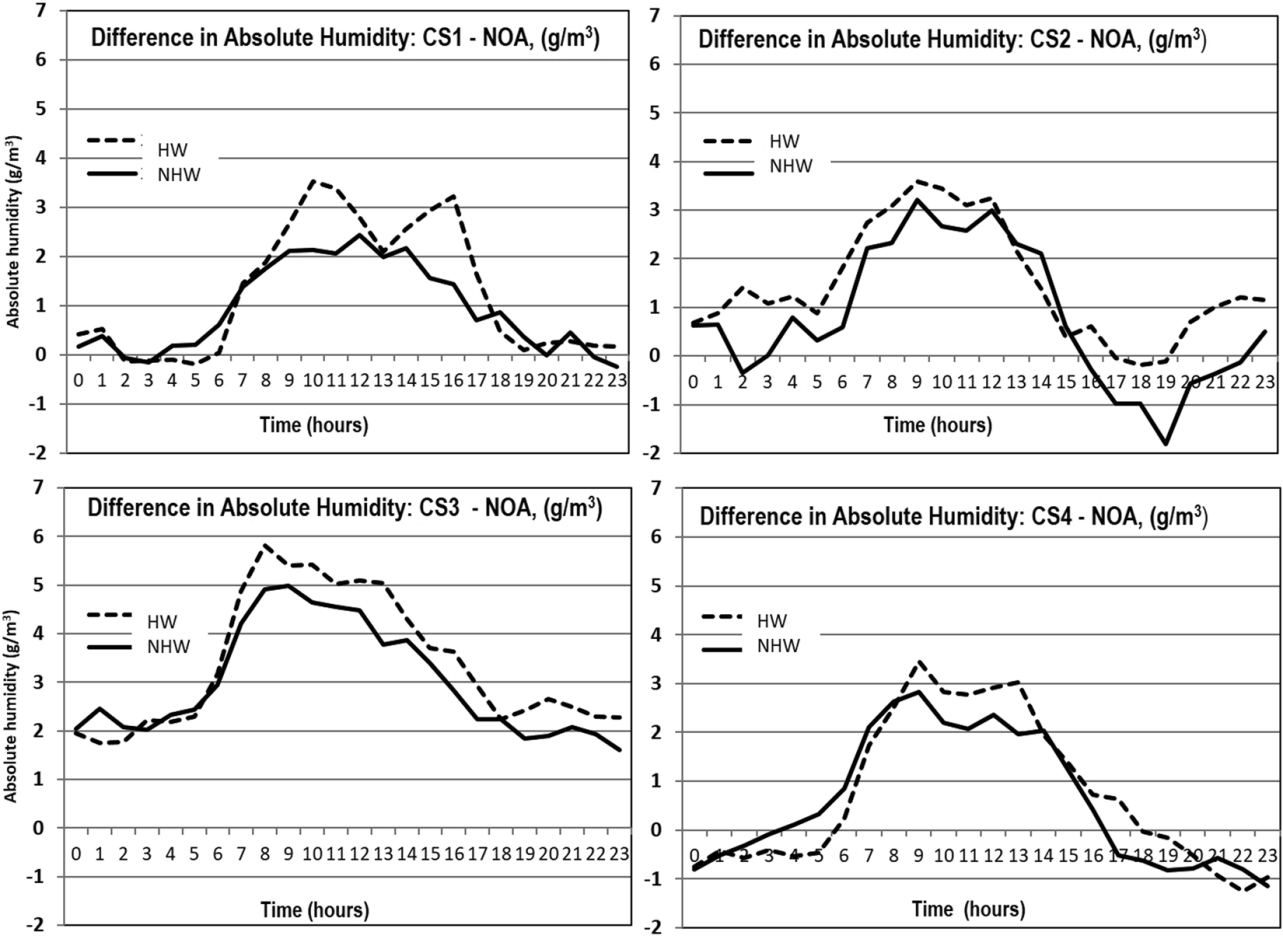
Diurnal patterns of the difference in the water content (g/m^3^) at each of the coastal stations and the urban one, during the HW (dashed line) and the NHW (solid line) periods.

To evaluate the potential difference of evapotranspiration between the considered urban and coastal areas during the HW and NHW periods, the levels of water content in the atmosphere (g/m^3^) were estimated and compared at all stations. Figure 5 shows the difference in atmospheric water content between each of the coastal stations and the urban one during both the HW and NHW periods. Water content at the coastal stations was found to be higher during the daytime for both periods. Higher water content at the coastal areas is attributed to the increased evaporation because of the proximity to the sea and possibly because of the advection of quite humid air transferred by the sea breeze. However, even during the days where the sea breeze did not develop and the air was advected from the mainland to the coast, the water content at the coastal sites was higher compared to the urban site. During the daytime of HW periods, the water content at the urban station was reduced on average by 4–5%, while it was enhanced by 5–6% in the coastal zones compared to the NHW periods. This resulted in an increased difference of the water content between the coastal and urban stations during the HW period (Fig. 5). This is attributed to: a) the reduced evaporation rate at the urban station because of the relatively drier ground surface, b) the increased or the constant evaporation rate at the coastal stations and c) the potential transfer of water vapor from the sea breeze circulation which prevailed during the HW period. The alteration of the evaporation rates in the coastal and the urban areas contribute to a further intensification of the urban heat island magnitude during the HW period. Lower latent heat fluxes in the urban than in the rural stations have also been reported during heat waves in Baltimore^39^ and Beijing^40^, contributing to synergies between HWs and UHIs.

The previous analysis focused on changes in the sensible and latent heat fluxes during HWs. Other terms in the surface energy budget equation might also contribute to synergies between HWs and UHIs; nevertheless, available measurements in our study cannot support a detailed analysis of each term in the budget equation at all sites. We further note here that previous studies on the topic highlighted the significance of the changes in the partition between sensible and latent heat fluxes during HWs, which are more instrumental in synergies between HWs and UHIs than changes in the total available energy^39, 40^.

### Nocturnal UHI

**Figure 6:**
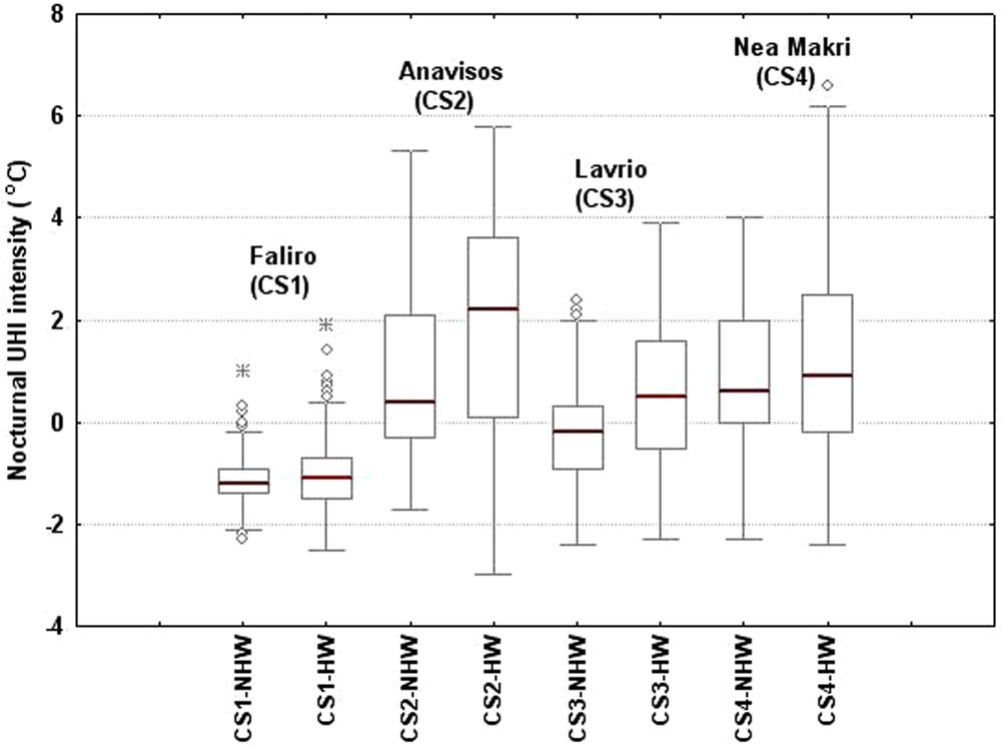
Box plots of hourly values of the nighttime UHI intensity at all coastal stations (CS1-CS4), during HW and NHW periods.

Νocturnal UHI intensity during HWs is of special interest, as the physiological impacts of high nighttime temperatures have been proven to be more important compared to high daytime temperatures^25, 38^. Lower pressure gradients during nighttime resulted in the deceleration of synoptic winds and prevalence of low winds at all sites, particularly during HW periods. Low wind speeds imply that advection mechanisms are not contributory to UHI intensity during nighttime and radiative processes mainly control the magnitude of the nocturnal UHI. Although cities cool less rapidly than surrounding rural areas resulting in pronounced nighttime UHIs^2–4^, the higher heat capacity of water at coastal sites establishes more stable conditions, moderating cooling rates after sunset. Figure 6 illustrates box plots of the nocturnal hourly values of UHI intensity at all coastal stations under HW and NHW conditions. Under NHW conditions, nocturnal UHI intensity is on average negative at CS1 and CS3 and slightly positive at CS2 and CS4, ranging around +1 °C. The radiative cooling rate at NOA under these conditions is slightly higher or comparable to radiative cooling at coastal stations, prohibiting the formation of a strong nocturnal UHI. Under HW conditions, the nocturnal UHI has a different behavior. Extra warmth gained by urban environment during the day is released during nighttime, retarding significantly or prohibiting cooling mechanism, thus keeping city very hot. Coastal stations are less affected due to more steady sea surface temperature, resulting to intensification of the nocturnal UHI. The nocturnal UHI intensity increases on average by up to 1.5 °C during HWs compared to NHW periods (maximum increase at CS2). The difference in the UHI intensity between HW and NHW conditions is less prominent at CS1, due to the location and high urban density of this station. It is noted however, that the nocturnal UHII exhibited a pronounced variability almost at all stations, possibly related also to other local factors.

## Discussion

Managing adverse effects of extreme heat events in urban areas requires a deep understanding of how the urban environment responds to extremely hot weather. This study enlightens some -still open- questions on the synergies between UHIs and HWs, focusing for the first time on coastal areas. Our results demonstrated that HWs intensify UHI during both daytime and nighttime. Two major factors regulated daytime UHI intensity during the study period: a) the daily maximum ambient temperature (T_*max*_) at the reference station and b) the wind field. The UHI intensity was found to increase with increasing T_*max*_, being always higher during HWs compared to NHW (summer background) conditions. Increased temperature during HWs was found to induce non symmetrical alterations in sensible and latent heat release between the urban and coastal sites, possibly contributing to synergies between HWs and UHI. The magnitude of the increase of UHI intensity during HWs was also highly dependent on the background wind field. Under conditions favorable for sea breeze development, a strong cooling mechanism at coastal stations induced pronounced intensification of the UHI which was higher on average by up to 3.5 °C compared to NHW conditions (and by up to 8 °C under specific conditions). The sensitivity of UHI intensity to wind direction implies the significance of horizontal advection fluxes at coastal sites and their contribution to the synergies between HWs and UHI.

HWs increased the nocturnal UHI intensity as well; nontheless, these changes were much less prominent compared to the daytime ones, due to the relatively low cooling rates at the coastal stations. Relevant studies report higher intensification (of the order of 2 °C) in the nocturnal rather than daytime UHI magnitude during HWs; however, comparisons are based on rural and not coastal surrounding stations^39, 40, 48^. Other studies report a slight intensification of daytime and nighttime UHI during hot days compared to average summer days^28^. Intensification only of the nocturnal UHI in London during the extreme HW of 2003 has been also reported^21^. Although lack of surface moisture and very low winds are usually considered to be behind the ‘added’ impact of HWs on UHI intensity^39^, our findings suggest synergies under a wider range of wind speeds. Up to now, the possible synergies have been explained in terms of the differences mainly in radiative processes and evapotranspiration rates, while advection phenomena are not analysed, although different responses of the wind field between urban and rural sites during HWs was recently reported in the Beijing Metropolitan Area^48^.

We found a synergetic relation between UHIs and HWs in the study area. Our findings are consistent between stations and different HW episodes. Moreover, the analysis is not based on historically very rare HWs (as for instance the record breaking HWs of 1987^30^ and 2007^49^ in the same area), but on HW episodes that are becoming increasingly more frequent. Additional experimental data are required in the future, to shed more light on the response of all components in the surface energy budget during HWs at urban and non urban sites and better understand feedbacks between UHIs and HWs.

Focused on a climatically and demographically sensitive area, we believe the study captures a phenomenon of high impact and the findings can be useful to understand the synergies between UHIs and HWs in cities with similar boundary and climatic conditions. Overall, the study proves a positive feedback between UHIs and HWs which not only is not negligible, but on the contrary can cause a jump in the UHI strength, highlighting the thermal comfort contrast between urban and non urban areas. In a warming and urbanizing world, the study points to the growing need for protecting the urban population by increasing awareness and preparedness against thermal risk.

## Methods

### Study area

Athens is a densely populated coastal area of the eastern Mediterranean, located at the southernmost part of the Greek mainland (see Fig. S1 in Supplementary). The city hosts approximately 3.8 million residents (including the suburbs) and ranks among the eight largest urban zones in Europe. Athens has been experiencing pronounced warming during the last decades, which in summer amounts to approximately +1^0^ C/decade since the mid 1970s, attributable to both global/regional warming and intensifying urbanization^41^. The increase in the mean air temperature is coupled with a simultaneous, vast increase in HW frequency^49^. The last decade has been marked by a number of record breaking heat related events, as for instance the highest temperature ever measured since the mid 19^th^ century (in 2007), the warmest summer ever recorded (in 2012), and the early heat waves in 2007, 2010 and 2016 (based on the historical 160-years long climatic records of NOA). The urban heat island is a well documented phenomenon in the city of Athens. Its intensity exhibits a strong spatial and temporal variability and becomes maximum during daytime in summer when, under favorable conditions, it can exceed 10 °C between rural areas and the central zone of the city^50^. Strong N-NE winds -known from antiquity as ‘Etesians’- are a common synoptic pattern for the area during summer. Etesian winds blow over the Aegean sea before entering the mainland and thus constitute a cooling mechanism for the city of Athens. Depending on their strength, ‘Etesians’ may block the formation of sea breezes from the Saronic Gulf (Fig. S1 in Supplementary), as they alter the local pressure difference that generates the sea breeze. Attenuation of ‘Etesians’ allows the onset of sea breeze circulation which develops mainly along the SW-NE axis at CS1 and CS2.

### Data

The period July-August 2012 was selected for analysis. Based on the historical climatic records of NOA, this period was hotter than any other corresponding period over at least the past 160 years in Athens, with +3.7^0^ C anomaly (corresponding to +3 standard deviations) in the daily maximum air temperature (T_*max*_) (with respect to the 1971–2000 climatic period). An unprecedented succession of consecutive HWs, five in total, occurred during the same period. Although two stronger HWs in terms of intensity and duration have hit the area in the past^30, 49^, this particular period has been considered quite challenging for study due to the continuous swing from HW to non HW conditions, but also the variety in the backround wind field. A set of fixed stations located within and around the Greater Athens Area (GAA) was used. The stations belong to the meteorological network of NOA and provide hourly data of near surface air temperature (at ~2 m a.g.l.), wind speed and direction, daily maximum and minimum air temperatures and relative humidity. The selected set includes four coastal stations (CS1, CS2, CS3 and CS4, established between 2009 and 2012) and the urban station of NOA. Locations and names of the stations are shown in Fig. S1 of Supplementary. Located in the centre of the city of Athens near the Acropolis, the historical climatic station of NOA bears the characteristics of an urban station, nevertheless, due to its position at an area of archaeological interest, the station is free from the direct effect of very high urban density, thus NOA has been considered ideal to represent a ‘reference’ urban station. Moreover, the NOA station has been proven to be representative of a larger scale, based on comparison with air temperature data over a number of other stations of the network, recently established at different distances within the city. The station CS1 is located at a suburb to the SW of Athens, thus it bears the characteristics of an urban coastal area. The other three coastal stations (CS2-CS4) are located in areas with low urban density. Coastal stations are well exposed and the same holds true for the urban station (NOA) which is located on a small hill, (~100 m a.s.l), in the centre of the city. The elevation of the stations is not very different, ranging between 3 and 100 m (CS1: 25 m, CS2: 10 m, CS3: 3 m and CS4: 90 m). Southerly winds are in general associated with advection of air masses from the sea (sea breezes) and northerly winds with advection from the mainland. The sea breeze at the stations CS1 and CS2- located to the west of the Attika peninsula (Fig. S1 in Supplementary)- develops mostly along the SW-NE axis. For the stations CS3 and CS4, winds blowing from SE to NE sectors correspond to air mass transfer from the sea.

Hourly values of air temperature and relative humidity were used for the estimation of the absolute humidity (water content in g/m^3^) at all stations^51^. MODIS (Moderate Resolution Imaging Spectroradiometer) satellite data of Land Surface Temperature at the areas of interest were extracted from the Aqua MODIS, MYD11A1.006, 1000 m, Daily product, https://lpdaacsvc.cr.usgs.gov/appeears).

### Definitions

A HW is defined as a sequence of at least three consecutive days with T_*max*_ > 37 °C. This temperature threshold value corresponds to the 95^th^ percentile of T_*max*_ distribution for the period of interest (based on the 1971–2000 climatic period at NOA)^52^. Coincidentally, it also lies within the temperature range of a healthy human (body temperature). We note here that defining a HW is still an open scientific issue and so far, adopted definitions in the literature vary with respect to the duration of the event, the selected climatic or bioclimatic indices or the threshold value to determine extremes^53^. The adopted definition in our analysis combines both relative (based on local climate) and absolute temperature thresholds related to the onset of physiological stress. As for the duration, this is in line with -or more strict than - other limits set worldwide, as for instance the least duration of 48 hours in USA^52^, or of three days in Australia and Europe^49, 54, 55^. A further discussion on HW definitions is beyond the scope of the present study. In our analysis, periods that satisfy the aforementioned criteria are registered as Heat Wave (‘HW’) periods, otherwise as non HW (‘NHW’) periods. According to the adopted definition, five HWs occurred in Athens from July 1^st^ to August 31^st^, 2012 (Fig. S2 in Supplementary). During three of them, air temperature peaked above 40^0^ C for three consecutive days. 30 days in total fall into HW periods and 32 days fall into NHW periods, ensuring almost equal size samples.

UHI intensity (UHII) was assumed as the ambient temperature difference (ΔT) between the urban station (NOA) and any coastal station, specified accordingly. A positive value indicates hotter conditions at NOA and a negative value cooler conditions. In the manuscript, the expression ‘UHI intensity at station X’ implies the ambient temperature difference between NOA and station ‘X’, namely T_(NOA)_ − T_(x)_.

### Surface energy budget

UHIs result from the modification of the energy balance in urban areas (compared to non urban areas) due to changes in surface materials, land use/land cover and additional human energy released in the urban environment. The energy budget equation is expressed as^56^

1 $${{\rm{Q}}}^{\ast }+{{\rm{Q}}}_{{\rm{F}}}={{\rm{Q}}}_{{\rm{H}}}+{{\rm{Q}}}_{{\rm{E}}}+{\rm{\Delta }}{{\rm{Q}}}_{{\rm{S}}}+{\rm{\Delta }}{{\rm{Q}}}_{{\rm{A}}}\quad ({{\rm{W}}/{\rm{m}}}^{2})$$

where Q* is the net all-wave radiative heat flux at the earth’s surface, Q_F_ the anthropogenic heating flux, Q_H_ and Q_E_ the turbulent sensible and latent heat fluxes respectively, ΔQ_S_ the storage heat flux and ΔQ_A_ the net heat flux by horizontal advection.

Equation (1) can be simplified to

2 $${{\rm{Q}}}^{\ast }={{\rm{Q}}}_{{\rm{H}}}+{{\rm{Q}}}_{{\rm{E}}}+{\rm{\Delta }}{{\rm{Q}}}_{{\rm{S}}}\quad ({\rm{W}}/{{\rm{m}}}^{2})$$

assuming that horizontal advection is not significant^39, 40, 57^. Anthropogenic heat flux is frequently omitted from the energy equation, as it is assumed that measurements of Q*, Q_H_, and Q_E_ are likely to include most of the anthropogenic contributions to the radiative, convective and conductive fluxes, thus already account for Q_F_ effects^57–59^. The sum of the turbulent sensible and latent heat fluxes is known as the ‘available energy’.

Quantification of the energy budget components at urban and non urban sites during HW and NHW periods would enlighten possible synergies between UHIs and HWs. Nevertheless, although the literature is very rich in studies concerning energy budget differences between urban and rural sites, relevant information regarding differences between urban and rural sites during HWs is missing, or is quite restricted to very few recent studies^39, 40, 48^. Unsymmetrical changes in sensible and latent heat fluxes but also wind speed between urban and rural sites have been reported to enhance UHII during HWs^39, 40, 48^.

In our study, changes in the available energy, i.e. sensible and latent heat fluxes between HW and NHW periods were approximated using satellite observations of land surface temperature and estimations of near-surface humidity at all sites (see Data). Changes in the partition of these fluxes during HWs have been proven to contribute to synergies between HWs and UHIs^39, 40^.

In the absence of relevant measurements (e.g. all-wave radiation) at all sites of interest, a detailed and comprehensive analysis of each term of the surface energy budget and investigation of differences between the urban and coastal sites is not feasible. Nevertheless, some interesting information concerning the reference station is provided below.

Analysis of short wave (SW) solar radiation measurements during the study period (available only at NOA) did not reveal any significant change in the incoming SW radiation between HW and NHW periods. Specifically, during the NHW period, the incoming SW radiation at NOA was found only slightly lower than that during the HW period, attributable to slightly increased cloudiness, mainly in the afternoon hours. (Fig. S3 in Supplementary). Due to the background climate of Athens, characterized by clear skies and dry conditions in summer, no significant changes in incoming SW radiation between HW and NHW periods were expected.

Estimation of anthropogenic heat flux is a difficult and quite challenging task since it depends on many factors and may vary considerably between cities. Different values ranging from 50 to 160 W/m^2^ during summer have been proposed for Athens^57, 60^, with cooling systems contributing by about 15–20 W/m^2, 59^. Moreover, analysis of the relation between the electricity demand and the ambient temperature in Athens, showed that the peak electricity load increases by about 4.1% per degree of temperature increase^61^. This corresponds to about 15–20% higher electricity demand during HWs (mainly due to the additional use of the cooling systems) and an additional anthropogenic heat flux of the order of 10 W/m^2^.

Previous experimental campaigns in Athens have demonstrated that storage heat flux ΔQ_S_ represent a high percentage of net radiation during summer in the city, with the ratio ΔQ_s_/Q* approximating or exceeding 0.5^57^. This value is quite consistent with findings in other cities with similar background climatic conditions (e.g. clear skies, dry period)^62^.

### Data availability

All data are available from the authors upon request.

## Electronic supplementary material

Founda&Santamouris-Supplementary
