# Supplementary material for: Synergies between Urban Heat Island and Heat Waves in Athens (Greece), during an extremely hot summer (2012)
Source: Sci Rep. 2017 Sep 8;7:10973. doi: 10.1038/s41598-017-11407-6 (PMC5591211; doi:10.1038/s41598-017-11407-6)
Supplement: Supplementary file 1 — Founda&Santamouris-Supplementary [file 41598_2017_11407_MOESM1_ESM.pdf]

Supplementary Information

Synergies between Urban Heat Island and Heat Waves in Athens (Greece), during an extremely hot summer (2012)

Dimitra Founda[1] and Mattheos Santamouris[2,3]

1. National Observatory of Athens, Institute for Environmental Research & Sustainable Development, Athens, Greece (founda@noa.gr)

2. The Anita Lawrence Chair in High Performance Architecture, School of Built Environment, University of New South Wales, Sydney, Australia

3. Physics Department, University of Athens, Greece.

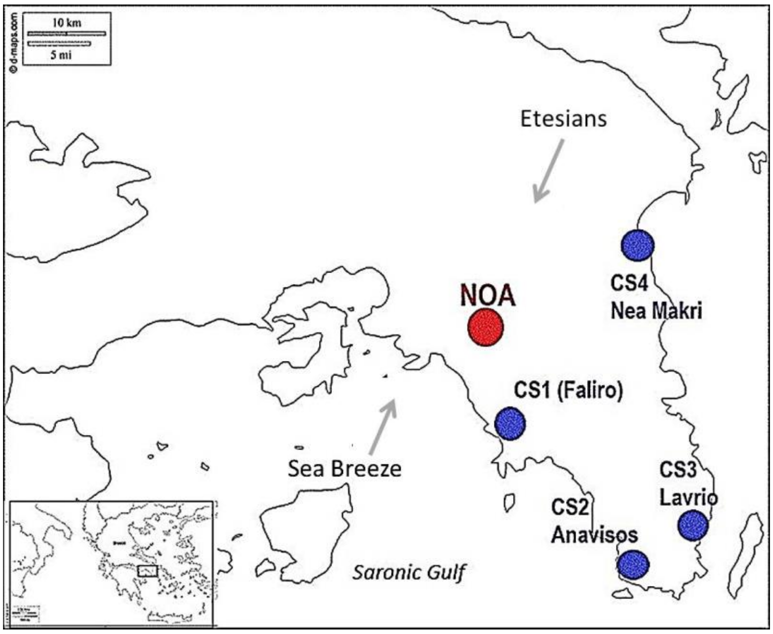

Figure S1. Map of the study area. The sites of the reference urban station NOA (red color) and the four coastal stations CS1 (Faliro), CS2 (Anavisos), CS3 (Lavrio) and CS4 (Nea Makri) (blue color) are shown on the map. Grey arrows indicate the prevailing wind directions corresponding to 'Etesians' synoptic pattern and sea breeze circulations.
(Maps obtained from *http://d-maps.com/carte.php?num_car=1946&lang=en* and *http://d-maps.com/carte.php?num_car=4982&lang=en*).

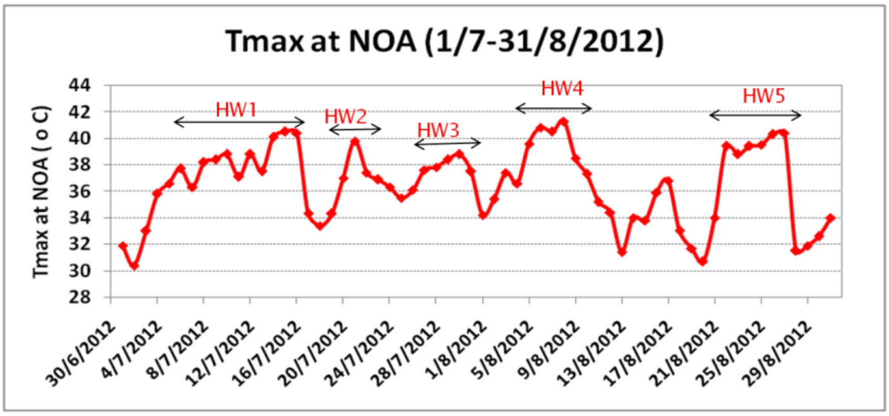

Figure S2: Evolution of the daily maximum ambient temperature (T_{max}) at the urban station (NOA) from July 1st to August 31st, 2012. Following the adopted definition of a heat wave (see Methods), five heat waves are discerned over this period.

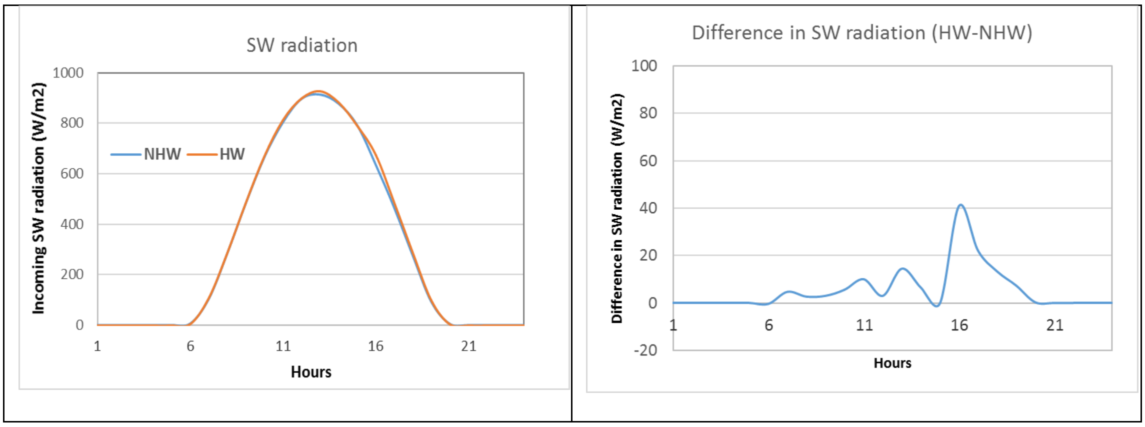

Figure S3. Average diurnal pattern of incoming short wave (SW) radiation at the reference station (NOA) during the HW and NHW periods (left) and their difference (right).

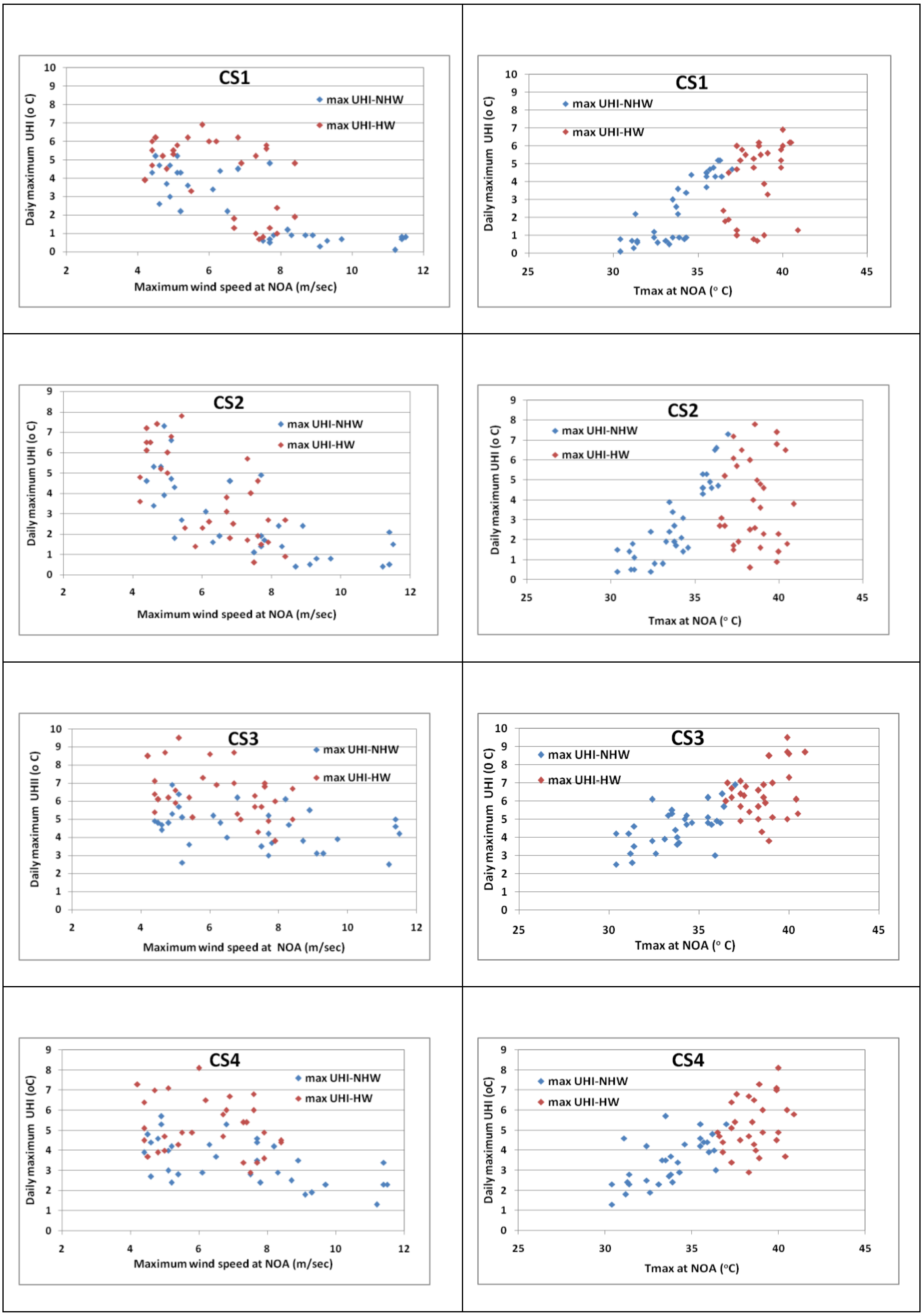

Figure S4. Variation of the daily maximum UHI intensity at stations CS1-CS4 as a function of the daily maximum wind speed (left panels) and T_{max} (right panels) at NOA, separating for HW and NHW conditions.
